# Heritable influences on behavioural problems from early childhood to mid-adolescence: evidence for genetic stability and innovation

**DOI:** 10.1017/S0033291715000173

**Published:** 2015-03-13

**Authors:** G. J. Lewis, R. Plomin

**Affiliations:** 1 Department of Psychology, University of York, Heslington, York, UK; 2 King's College London, MRC Social, Genetic and Developmental Psychiatry Centre, Institute of Psychiatry, Psychology & Neuroscience, De Crespigny Park, London, UK

**Keywords:** Behavioural problems, genetics, longitudinal, SDQ, twin study

## Abstract

**Background:**

Although behavioural problems (e.g. anxiety, conduct, hyperactivity, peer problems) are known to be heritable both in early childhood and in adolescence, limited work has examined prediction across these ages, and none using a genetically informative sample.

**Method:**

We examined, first, whether parental ratings of behavioural problems (indexed by the Strengths and Difficulties questionnaire) at ages 4, 7, 9, 12, and 16 years were stable across these ages. Second, we examined the extent to which stability reflected genetic or environmental effects through multivariate quantitative genetic analysis on data from a large (*n* > 3000) population (UK) sample of monozygotic and dizygotic twins.

**Results:**

Behavioural problems in early childhood (age 4 years) showed significant associations with the corresponding behavioural problem at all subsequent ages. Moreover, stable genetic influences were observed across ages, indicating that biological bases underlying behavioural problems in adolescence are underpinned by genetic influences expressed as early as age 4 years. However, genetic and environmental innovations were also observed at each age.

**Conclusion:**

These observations indicate that genetic factors are important for understanding stable individual differences in behavioural problems across childhood and adolescence, although novel genetic influences also facilitate change in such behaviours.

## Introduction

Twin and family studies have indicated that behavioural problems in childhood (Saudino *et al.* 2005) and adolescence (Scourfield *et al.* 2004) are underpinned by substantial genetic influences. However, less is known about the genetic stability of these influences. It is possible that genetic influences are broadly stable across these ages, reflective of an early maturing biological basis to behavioural problems that endures throughout childhood and adolescence. By contrast, the heritable effects at each of these ages may reflect genetic innovation, with novel heritable effects becoming apparent over the course of development, perhaps reflecting prolonged neural maturation (Gogtay *et al.* 2004; Blakemore & Choudhury, 2006). An intermediate position, positing that stable genetic effects are evident over childhood and adolescence accompanied by novel genetic effects emerging over time, is also plausible. Determining which of these perspectives best characterizes the genetic architecture of behavioural problems over childhood and adolescence will be important in order to better understand the manner in which gene effects exert their influences on behavioural problems. In addition, if genes are to be discovered for child and adolescent behavioural problems it is critical to know whether the same genetic influences are apparent across these stages in life. To address these issues, the current study used a longitudinal twin design in order to investigate the extent to which genetic influences contribute to the stability and innovation of genetic and environmental effects on a set of core behavioural problems.

### Child and adolescent behavioural problems

Understanding the aetiology of adolescent behavioural problems – such as problems with peer relationships, hyperactivity, conduct, and anxiety, and (low) prosociality – is of importance as such behaviours show links to broad-based life and psychosocial outcomes (Rutter, 1995). Research has suggested that around one in five adolescents satisfy diagnostic criteria for at least one psychiatric disorder (Merikangas *et al.* 2010), with specific behaviour problems such as conduct disorder, hyperactivity disorder, and anxiety/emotional problems all relatively common among adolescents (Ford *et al.* 2003; Merikangas *et al.* 2010). These behavioural problems are of clear and broad interest because of their links to mental health, school achievement, antisocial behaviour, and delinquency (Rutter, 1995; Aunola *et al.* 2000; Fergusson *et al.* 2005).

Several measurement instruments exist to screen for such behaviours (Achenbach, 1991*a*–*c*), including – as well as being the focus of the current study – the Strengths and Difficulties Questionnaire (SDQ; Goodman, 1997). The SDQ is a brief dimensional measure that covers the core domains of child and adolescent psychopathology (conduct problems, hyperactivity-inattention, emotional symptoms/anxiety, and peer relationship problems) alongside personal strengths (prosociality).

### Genetic origins of behavioural problems

Research examining the aetiology of such problem behaviours has indicated a role for genetics. For instance, children's and adolescents’ scores (ages 5–17 years) on the conduct problems scale of the SDQ have been reported to be substantially heritable (35–77%) (Scourfield *et al.* 2004). Similarly high heritabilities were found for all of the SDQ scales at age 7 years (Saudino *et al.* 2005) and age 16 years (Lewis *et al.* 2014). Moderate to substantial heritabilities (⩾0.40) have also been found for related measures in childhood and adolescence, such as antisocial behaviour and callous-unemotional traits (Viding *et al.* 2005) and internalizing and externalizing behaviours (Bartels *et al.* 2004). In sum, child and adolescent behaviour problems across multiple domains exhibit moderate-to-large heritable effects.

### Temporal stability of genetic effects

Less clear at this stage is whether these heritable influences on behavioural problems represent stable and enduring effects or instead are more transient and time-specific. While a number of phenotypically oriented studies have noted that a range of temperaments and behaviours (both normal and disordered) in adolescence and adulthood can be predicted by measures in early childhood (e.g. Caspi & Silva, 1995; Caspi *et al.* 1996; Hampson & Goldberg, 2006; Slutske *et al.* 2012), thus illustrating the temporal stability of behavioural problems, less work has addressed this question using a genetically informative design. Of work addressing SDQ domains, evidence for genes influencing both stability and innovation has been reported for prosociality from ages 2–7 (Knafo & Plomin, 2006) and for anxiety from ages 7–9 (Trzaskowski *et al.* 2012). More broadly, a study of externalizing and internalizing behaviours across four time-points from ages 3–12 found that a transmission model fitted the data best, such that genetic influences were stable over time but that novel genetic influences were apparent at each measured age (Bartels *et al.* 2004). More recent work found broad support for this finding, noting genetic stability on externalizing (Wichers *et al.* 2013), antisociality (Tuvblad *et al.* 2011), and attention problems (Chang *et al.* 2013) from ages 8–19, although again novel genetic influences were also observed for each of these variables over this time period. Broadly consistent findings have been reported for common childhood fears (e.g. snakes, spiders; Kendler *et al.* 2008).

Some reported findings, however, have diverged from this pattern of genetic stability. For example, a study examining conduct and delinquency reported that the genetic influences evident in early childhood were dissociable from those acting in mid-adolescence (Van Hulle *et al.* 2009). This observation (as the authors note) may have emerged as an artifact of the study design – parental reports were used in early childhood, self-report was used in adolescence; but as this study is unique in its examination the time-period from early childhood through to adolescence the findings raise important questions concerning the nature of genetic stability in behavioural problems across development.

### The current study

While research in recent years has begun to establish that heritable influences on different aspects of behavioural problems are fairly stable across broad stretches of development – most notably from age 10 onwards – specific questions remain unanswered. First, as noted above, the sole study to address stability from early childhood into adolescence reported that antisocial behaviour was underpinned by genetic influences at ages 4–9 which were wholly distinct to those genetic effects acting on antisocial behaviour at ages 14–17. Moreover, it is unclear if a similar pattern of stability and change exists for broader components of behavioural problems, such as those indexed by the SDQ. More generally, while important longitudinal genetically informative research has been focused on behavioural problems, no longitudinal genetically informative work has directly addressed prosociality and peer problems across from early childhood into adolescence, and work addressing anxiety and hyperactivity, while of value in its own right, has largely focused at the aggregated level of internalizing and externalizing (e.g. Bartels *et al.* 2004; Wichers *et al.* 2013).

A better understanding of the nature of genetic influences on behavioural problems over this important period of development is important for several reasons. First, knowledge of the dynamics of genetic influences on behavioural problems will serve to more accurately characterize the origins of behavioural problems. Second, environmental influences, and specifically non-shared-environment effects, are known to be large for behavioural problems, but this variance component also includes measurement error and so it is unclear whether this aetiology reflects a stable influence on behavioural problems or is better understood as a more transient factor. Third, if distinct sets of genes underpin behavioural problems at different ages, locating the causal variants will be significantly more challenging if researchers aggregate samples across ages. As such, knowledge of the genetic stability of behavioural problems across these ages will inform gene discovery strategies on whether aggregation across ages is a valid approach.

To rectify these gaps of knowledge in the literature, here we examined the extent to which genetic influences on childhood and adolescent behavioural problems are underpinned by stable genetic effects present at each measured age, or are more accurately described by genetic effects specific to each developmental age. We used the SDQ instrument to tap behavioural problems with parental scores taken at each age in order to maintain a consistent mode of rating across each wave of measurement. Ratings were available for ages 4, 7, 9, and 12 for all SDQ scales, and also for age 16 for conduct, hyperactivity, and prosociality.

## Method

### Participants

Participants were drawn from the Twins Early Development Study (TEDS), which is an ongoing longitudinal study following monozygotic (MZ) and dizygotic (DZ) twins born in England and Wales between 1994 and 1996 (Oliver & Plomin, 2007). The TEDS sample is representative of the UK population (Kovas *et al.* 2007) and the project received approval from the Institute of Psychiatry Ethics Committee. Twin zygosity was determined using a parental rating measure of similarity and DNA genotyping (Price *et al.* 2000). The number of complete twin pairs at each age were as follows: MZ male pairs: *n* = 533–1200; MZ female pairs: *n* = 670–1368; DZ male pairs: *n* = 505–1198; DZ female pairs: *n* = 555–1255; and DZ opposite-sex pairs: *n* = 996–2355.

### Measures

#### SDQ

The SDQ is a short but reliable instrument (25 items; Goodman, 2001; Stone *et al.* 2010) for measuring psychosocial problems in children (Goodman, 1997). The SDQ consists of five scales measuring anxiety, conduct problems, hyperactivity-inattention, peer problems, and prosocial behaviour. Scores for all subscales were acquired by parental rating when the child was 4, 7, 9, and 12 years old. Scores for prosociality, conduct, and hyperactivity were also acquired by parental rating when the individual was 16 years old. Cronbach's *α* was low (<0.60) for conduct (all ages) and for peer problems (ages 4 and 7), although in line with previously reported values (Goodman, 2001), but broadly acceptable for the rest of the SDQ measures.

### Analysis

Correlations between twins differing in their degrees of genetic relatedness (e.g. MZ and DZ twins) are informative as a guiding heuristic to estimate relative magnitudes of genetic and environmental effects (Plomin *et al.* 2013). The presence of genetic effects is inferred if correlations between MZ twins are larger than correlations for DZ twins. The presence of shared-environment effects is inferred if correlations for DZ twins are larger than half the magnitude of the correlations for MZ twins. Finally, non-shared-environment effects are inferred if correlations for MZ twins are less than 1.0, and so this variance component also contains measurement error. These correlation analyses were extended using formal model-fitting of variance-covariance matrices for the twin data. This approach allows parameter estimates in univariate models to be formally tested for significance as well as allowing multivariate models – the core focus of the current study – to be analysed.

**Fig. 1.**
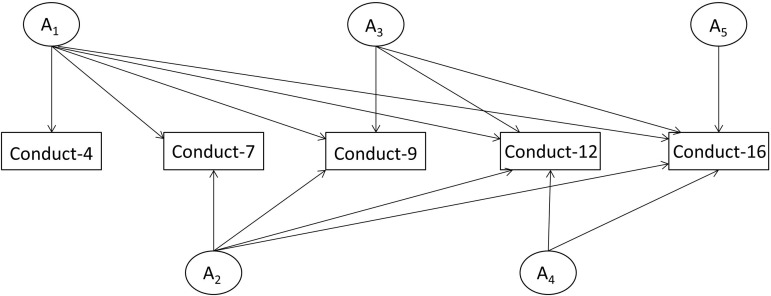
A figurative representation of the multivariate Cholesky (for conduct ages 4–16).

In the current study, longitudinal analyses were central to our tests. We sought to determine the extent to which genetic effects underlying SDQ measures across the ages reflected stable *v.* novel genetic influences. To perform this analysis, we compared four classes of model: the Cholesky decomposition, the common pathway model, the independent pathway model, and the simplex/transmission model, which are each detailed below (see Fig. 1 and online Supplementary Figs S1–S3).

The Cholesky decomposition specifies as many factors as there are variables for each source of variance, with each subsequent factor having one fewer pathway than the preceding factor (see Fig. 1). In other words, for additive genetic effects (A) the first latent factor loads on all of the *n* measured variables. The subsequent latent factors load on *n*_−1_, *n*_−2_, …, *n*_−*i*_ variables. In this way each factor accounts for as much of the remaining variance as possible, until the last factor accounts for just the remaining variance in the last measured variable. This is repeated for the shared-environment factors (C) and non-shared-environment factors (E). This design can also be viewed as allowing the genetic and environmental influences explaining SDQ measures at age 4 to also explain SDQ measures at the later ages, while also leaving specific genetic and environmental paths available to explain SDQ variation at the later ages that does not covary with SDQ measures at age 4.

The common pathway model is a more restricted model, constraining all genetic and environmental variance though a single pathway (Kendler *et al.* 1987) (see online Supplementary Fig. S1). The independent pathway model also constrains genetic and environmental covariation, but instead of requiring all sources of this covariance to be channelled through a single pathway, this model allows for independent general effects of genes, shared environments, and non-shared environments (see online Supplementary Fig. S2). Finally, the simplex/transmission model estimates genetic and environmental effects specific to each age, with these effects also allowed to ‘carry-over’ from one age to the subsequent age (see online Supplementary Fig. S3).

## Results

Descriptive statistics for all measures are presented in Table 1. Assumption testing using all twins indicated that means and variances could be equated across twin order, zygosity, and sex for most variables, with the small number of significant differences observed consistent with the large number of tests performed. Of note, however, was evidence for modest-to-moderate mean sex differences, particularly for hyperactivity and prosociality (see Table 1 and text below for more detail). Sex-limitation modelling (testing for quantitative and qualitative genetic and environmental differences across sex) largely indicated that genetic and environmental influences could be equated across sex, with the significant differences that were observed being either small in magnitude or, again, consistent with the large number of tests performed. Following these observations we pooled our sample across sex, but used sex-residualized variables.

**Table 1.** Descriptive statistics for Strengths and Difficulties Questionnaire (SDQ) subscales

Measure	*α*	MZm	MZf	DZm	DZf	DZosm	DZosf
SDQ (age 4 yr)							
Anxiety	0.60	1.28 (1.35)	1.41 (1.43)	1.39 (1.44)	1.49 (1.49)	1.33 (1.40)	1.32 (1.43)
Conduct	0.54	2.26 (1.58)	1.91 (1.46)	2.27 (1.60)	1.97 (1.54)	2.16 (1.60)	1.88 (1.50)
Hyperactivity	0.76	4.37 (2.24)	3.74 (2.07)	4.21 (2.44)	3.77 (2.36)	4.38 (2.36)	3.32 (2.18)
Peer problems	0.47	1.40 (1.41)	1.23 (1.34)	1.70 (1.57)	1.45 (1.48)	1.63 (1.57)	1.37 (1.42)
Prosociality	0.69	7.04 (1.84)	7.56 (1.78)	7.11 (1.90)	7.61 (1.81)	7.09 (1.92)	7.71 (1.77)
SDQ (age 7 yr)							
Anxiety	0.63	2.02 (1.76)	2.37 (1.84)	2.05 (1.78)	2.31 (1.88)	2.02 (1.77)	2.25 (1.83)
Conduct	0.59	1.88 (1.69)	1.50 (1.47)	1.94 (1.74)	1.55 (1.56)	1.79 (1.71)	1.48 (1.52)
Hyperactivity	0.76	3.99 (2.56)	3.18 (2.30)	3.90 (2.60)	3.34 (2.47)	4.13 (2.66)	2.89 (2.35)
Peer problems	0.59	0.90 (1.31)	0.76 (1.20)	1.17 (1.54)	0.95 (1.32)	1.13 (1.55)	0.93 (1.33)
Prosociality	0.67	7.98 (1.78)	8.52 (1.58)	7.85 (1.88)	8.44 (1.63)	7.87 (1.85)	8.56 (1.53)
SDQ (age 9 yr)							
Anxiety	0.69	1.51 (1.78)	1.94 (2.00)	1.52 (1.80)	1.93 (1.94)	1.51 (1.74)	1.77 (1.90)
Conduct	0.59	1.48 (1.54)	1.07 (1.33)	1.47 (1.52)	1.16 (1.31)	1.35 (1.49)	1.13 (1.32)
Hyperactivity	0.77	3.81 (2.46)	2.86 (2.06)	3.51 (2.51)	2.89 (2.21)	3.82 (2.59)	2.58 (2.06)
Peer problems	0.68	0.95 (1.39)	0.85 (1.36)	1.25 (1.74)	1.08 (1.49)	1.18 (1.62)	0.94 (1.35)
Prosociality	0.69	7.92 (1.87)	8.70 (1.43)	7.90 (1.81)	8.58 (1.58)	7.92 (1.77)	8.59 (1.53)
SDQ (age 12 yr)							
Anxiety	0.68	1.65 (1.80)	1.92 (1.97)	1.65 (1.84)	1.93 (1.94)	1.65 (1.86)	1.89 (1.97)
Conduct	0.57	1.43 (1.45)	1.16 (1.34)	1.49 (1.56)	1.21 (1.41)	1.40 (1.53)	1.23 (1.40)
Hyperactivity	0.77	3.35 (2.25)	2.29 (1.96)	3.23 (2.39)	2.50 (2.13)	3.48 (2.49)	2.14 (1.87)
Peer problems	0.64	1.11 (1.50)	0.88 (1.28)	1.23 (1.64)	1.04 (1.44)	1.27 (1.66)	0.96 (1.36)
Prosociality	0.67	8.31 (1.72)	8.83 (1.50)	8.20 (1.74)	8.84 (1.49)	8.28 (1.73)	8.78 (1.49)
SDQ (age 16 yr)							
Conduct	0.52	1.16 (1.31)	1.12 (1.28)	1.25 (1.34)	1.23 (1.43)	1.26 (1.45)	1.16 (1.35)
Hyperactivity	0.71	2.45 (2.01)	1.88 (1.65)	2.53 (2.03)	2.06 (1.99)	2.78 (2.19)	1.84 (1.71)
Prosociality	0.73	8.00 (1.95)	8.53 (1.86)	7.91 (2.01)	8.56 (1.78)	7.93 (2.01)	8.46 (1.81)

MZ, Monozygotic; DZ, dizygotic; m, male; f, female; os, opposite sex.

*α*, Cronbach's *α* for scale scores collapsed across sex and zygosity.

Values given are mean (standard deviation).

### Phenotypic associations

Zero-order correlations across age for the SDQ subscales are shown in Table 2. In all cases significant associations were noted with correlations between ages 4–16 ranging from 0.25 to 0.31. These associations indicate a moderate degree of phenotypic stability for behavioural problems across childhood and into adolescence.

**Table 2.** Zero-order correlations among Strengths and Difficulties Questionnaire (SDQ) subscales from ages 4–16

Measure	4 years	7 years	9 years	12 years
Anxiety_7 years_	0.38			
Anxiety_9 years_	0.38	0.51		
Anxiety_12 years_	0.31	0.45	0.51	
Anxiety_16 years_	–	–	–	–
Conduct_7 years_	0.47			
Conduct_9 years_	0.43	0.55		
Conduct_12 years_	0.40	0.52	0.56	
Conduct_16 years_	0.28	0.37	0.39	0.48
Hyperactivity_7 years_	0.54			
Hyperactivity_9 years_	0.51	0.65		
Hyperactivity_12 years_	0.43	0.57	0.64	
Hyperactivity_16 years_	0.31	0.41	0.45	0.55
Peer problems_7 years_	0.29			
Peer problems_9 years_	0.26	0.45		
Peer problems_12 years_	0.24	0.41	0.48	
Peer problems_16 years_	–	–	–	–
Prosociality_7 years_	0.41			
Prosociality_9 years_	0.39	0.49		
Prosociality_12 years_	0.33	0.44	0.51	
Prosociality_16 years_	0.25	0.33	0.37	0.44

One randomly chosen member from each twin pair, *n* = 1886–5653.

All correlations are *p* < 0.001.

### Multivariate twin analyses

We next assessed genetic and environmental links from SDQ measures across ages. We fitted each of our models individually for each SDQ scale using all available ages. In each case, the common pathway, independent pathway, and simplex models provided a significantly poorer fit to the data compared to the Cholesky (see online Supplementary Table S1) and so we retained the Cholesky as our final model for each of the SDQ measures (see Tables 3 and 4). Several key points are notable from these final models. First, heritable influences on all of the SDQ measures at age 4 are stable across all subsequent ages. Second, genetic innovation was also apparent, with significant novel heritable influences apparent at each age, and these novel heritable effects were in almost all cases stable over subsequent ages.

**Table 3.** Multivariate (Cholesky) modelling results for Strengths and Difficulties Questionnaire (SDQ) subscales (prosociality, conduct, and hyperactivity) from ages 4–16

	a1	a2	a3	a4	a5	c1	c2	c3	c4	c5	e1	e2	e3	e4	e5
Conduct_4 years_	**0**.**77**					**0.23**					**0.60**				
Conduct_7 years_	**0.47**	**0.63**				**0.32**	**0.18**				**0.09**	**0.48**			
Conduct_9 years_	**0.40**	**0.29**	**0.53**			**0.41**	−0.10	**0.29**			**0.05**	**0.16**	**0.43**		
Conduct_12 years_	**0.37**	**0.33**	**0.28**	**0.48**		**0.38**	−0.17	−0.20	0.00		**0.05**	**0.09**	**0.14**	**0.44**	
Conduct_16 years_	**0.35**	**0.24**	**0.15**	**0.28**	**0.64**	**0.17**	−0.05	0.09	0.00	0.00	0.02	**0.05**	**0.08**	**0.10**	**0.50**
															
Hyperactivity_4 years_	**0.60**					0.00					**0.80**				
Hyperactivity_7 years_	**0.47**	**0.52**				0.00	0.00				**0.33**	**0.63**			
Hyperactivity_9 years_	**0.54**	**0.33**	**0.53**			0.00	0.00	0.00			**0.23**	**0.28**	**0.44**		
Hyperactivity_12 years_	**0.52**	**0.31**	**0.23**	**0.56**		0.00	0.00	0.00	0.00		**0.15**	**0.22**	**0.18**	**0.41**	
Hyperactivity_16 years_	**0.46**	**0.25**	**0.13**	**0.25**	**0.62**	0.00	0.00	0.00	0.00	0.00	**0.07**	**0.14**	**0.11**	**0.10**	**0.47**
															
Prosociality_4 years_	**0.72**					**0.25**					**0.64**				
Prosociality_7 years_	**0.50**	**0.61**				−0.11	0.14				**0.09**	**0.58**			
Prosociality_9 years_	**0.35**	**0.42**	**0.52**			**0.36**	0.15	0.27			**0.06**	**0.18**	**0.41**		
Prosociality_12 years_	**0.30**	**0.33**	**0.30**	**0.57**		**0.21**	0.14	−0.24	0.18		**0.04**	**0.14**	**0.16**	**0.44**	
Prosociality_16 years_	**0.27**	**0.19**	**0**.06	**0.33**	**0.59**	**0.17**	**0.43**	0.06	−0.25	0.05	0.01	**0.06**	**0.09**	**0.05**	**0.37**

a, Additive genetic effects; c, shared-environment effects; e, non-shared-environment effects.

Standardized path coefficients are reported.

Bold values indicate that the confidence intervals (95%) did not cross zero.

**Table 4.** Multivariate (Cholesky) modelling results for Strengths and Difficulties Questionnaire (SDQ) subscales (peer problems and anxiety) from ages 4–12

	a1	a2	a3	a4	c1	c2	c3	c4	e1	e2	e3	e4
Anxiety_4 years_	**0**.**69**				**0.28**				**0.66**			
Anxiety_7 years_	**0.38**	**0.55**			**0.25**	**0.32**			**0.09**	**0.61**		
Anxiety_9 years_	**0.32**	**0.25**	**0.54**		**0.41**	0.04	0.12		**0.04**	**0.23**	**0.55**	
Anxiety_12 years_	**0.33**	**0.31**	**0.23**	**0.51**	**0.27**	−0.02	−0.10	0.00	**0.03**	**0.15**	**0.19**	**0.59**
Peer problems_4 years_	**0.81**				0.15				**0.56**			
Peer problems_7 years_	**0.32**	**0.71**			0.04	0.12			**0.10**	**0.61**		
Peer problems_9 years_	**0.32**	**0.46**	**0.57**		0.14	−0.25	0.00		0.03	**0.16**	**0.50**	
Peer problems_12 years_	**0.27**	**0.35**	**0.31**	**0.65**	0.16	0.03	0.00	0.00	**0.04**	**0.14**	**0.13**	**0.48**

a, Additive genetic effects; c, shared-environment effects; e, non-shared-environment effects.

Standardized path coefficients are reported.

Bold values indicate that the confidence intervals (95%) did not cross zero.

While levels of innovation were broadly mirrored for non-shared-environment influences, the stability of non-shared-environment influences was considerably more modest for each measure. Finally, shared-environment effects were significant, albeit modest in magnitude, for anxiety, conduct, and prosociality, at age 4. These effects were broadly stable over subsequent ages. Some evidence for novel shared-environment effects was also observed – namely, anxiety at age 7 and conduct at ages 7 and 9 – although these effects were typically modest.

## Discussion

The current study provides several important insights into the aetiology of childhood and adolescent behavioural problems. At the phenotypic level we saw significant prediction across childhood and adolescence such that scores on the SDQ scales at age 16 (age 12 for anxiety and peer problems) were predicted by the corresponding SDQ score at age 4. These phenotypic overlaps were mostly accounted for by genetic factors, although evidence for modest shared environmental stability was observed (with the exception of hyperactivity). While research has suggested that genetic influences on antisociality shows distinct genetic underpinnings in early childhood *v.* early adolescence (Van Hulle *et al.* 2009), the current results using parental report across all measured ages (Van Hulle *et al.* relied on different raters in childhood *v.* adolescence) indicates that aspects of antisociality (i.e. conduct, hyperactivity), as well as broader elements of behavioural problems, are genetically stable from as early as age 4, in line with longitudinal work examining such phenotypes from later in childhood (e.g. Wichers *et al.* 2013). We also noted substantial genetic innovation at each age for each of the SDQ measures, which in turn were then seen to remain stable across the following ages. Environmental influences, and specifically non-shared-environment effects, largely acted to provide time-specific sources of variance.

These findings converge with related work stressing that behavioural measures taken in early childhood predict later life psychopathology (Caspi *et al.* 1996; Slutske *et al.* 2012), although these effects tend to be modest-to-moderate when spanning substantial time ranges and the current results are consistent with such observations. The current results also converge with recent research demonstrating gene variants linked to risk for psychopathology in adults predict neuroanatomical variation in infants (Knickmeyer *et al.* 2014) and suggest that individual differences in measurable neurophysiology (such as regional grey- or white-matter structure) in early childhood may also serve as a useful biomarker for subsequent behavioural problems. Our observation that behavioural problems are underpinned by novel heritable influences across development is also in line with recent work demonstrating heritable effects on cortical development operate in similar fashion (Schmitt *et al.* 2014). Assuming that cortical maturation mediates the impact of novel genetic influences on behavioural problems raises important questions regarding the brain bases of the early *v.* later emergent genetic bases of behavioural problems. Given that brain regions governing high-level cognitive control, such as the dorsolateral prefrontal cortex, appear to mature comparatively late in adolescence (Gogtay *et al.* 2004; Blakemore & Choudhury, 2006), one possibility is that the novel genetic influences on behavioural problems, particularly those reflecting aspects of disinhibition or poor impulse control (e.g. hyperactivity), may reflect genetic influences on the latter-stages in development of these neural structures.

Specific recommendations for future research are warranted. First, from a methodological standpoint, the degree of genetic innovation observed across the ages suggests that gene discovery should either focus on adult individuals, where genetic innovation is less evident (Johnson *et al.* 2005), or samples of children of equal age. Second, we relied entirely on parental report data. While this approach had the advantage of allowing us to use the same source of measurement across multiple ages, multiple raters would have potentially provided a more accurate assessment of the individual being rated. In particular, parental ratings may result in underestimates of heritability and overestimates of shared environment. While we cannot rule out this possibility, here we observed high heritabilities across all measures, with only modest shared-environment effects, suggesting that this possible source of bias had no major effect on our estimates. Third, the classical twin design is subject to a number of assumptions, such as the equal environment assumption (Neale & Cardon, 1992). Future studies that can capitalize on additional family structures in order to provide more assumption-free estimates would be valuable, although it is noteworthy that research testing whether violations of the equal environment assumption are apparent for psychopathology has found little evidence for this potential source of bias (Kendler *et al.* 1993; Derks *et al.* 2006). Finally, genetically informative latent growth modelling may help to further delineate the factors underlying developmental changes in behavioural problems and so would be a valuable direction to follow in future research.

In summary, here we report for the first time evidence that genetic influences on behavioural problems in early childhood are stable across childhood and into adolescence. Of importance, we also observed novel genetic influences at each age, with non-shared-environment effects also providing additional novel sources of variance across this important developmental phase. These findings support models of behavioural problems that posit early emerging and enduring individual differences in biology reflect such phenotypes, although also suggesting that the genetic influences underlying behavioural problems unfold across childhood and adolescence.

## Supplementary material

For supplementary material accompanying this paper visit http://dx.doi.org/10.1017/S0033291715000173.
